# Juvenile idiopathic arthritis is a diagnosis of exclusion

**DOI:** 10.1186/1546-0096-12-S1-P224

**Published:** 2014-09-17

**Authors:** Ekaterina Alexeeva, Rina Denisova, Sanya Valieva, Tatyana Bzarova, Ksenya Isayeva, Tatyana Sleptsova, Evgeniya Chistyakova, Anna Fetisova, Olga Lomakina

**Affiliations:** 1 Rheumatology, Scientific Center of Children's Health of RAMS, Moscow, Russian Federation

## Introduction

Juvenile Idiopathic Arthritis (JIA) is classified as an acquired autoinflammatory disease. Problems in applying all classification criteria include the necessity to exclude other diseases with similar symptoms. There are no laboratory markers for sJIA.

## Objectives

To analyze cases with cancer diagnosis who were in rheumatology department in SCCH of RAMS.

## Methods

13 patients were included in the analysis. All Patients were directed (referred) to our clinic like patients who were suffered from JIA from 2004 to 2014 years. We analyzed time concomitant therapy, symptoms of diseases, cancer diagnosis, diagnostic methods which helped to exclude JIA.

## Results

11 patients were directed with systemic JIA and 2 - with olygoarthritis. All patients with “JIA” had criteria for diagnosis according ILAR classification. All patients with ''systemic JIA'' had fever, hepatosplenomegaly, generalized lymphadenopathy, arthralgia or arthritis, high level of CRP, ESR, anemia. Patients with ''óligoarthritis'' had monoarthritis. The diagnosis was made at the place of residence. 6 patients received immunodepressants (methotrexate, cyclosporine), 8 patients – glucocoticoids, 1 – tocilizumab. Disease duration was from 2 to 9 months, the duration of hospitalization until the diagnosis was verified was from 1 to 7 days. We performed ultrasound, radiography, CT scan, MRI, bone marrow punction, biopsy of lymph node, biopsy of bone. There were 4 patients with neuroblastoma, 2 - with lymphogranulomatosis, 4 - with leukemia, 1- with malignant lymphoma from patients with “sJIA”. There was 1 patient with malignant histiocytoma and 1 patient with glyoma from patients with “oligoarthritis”. Concomitant treatment made difficult to verify right diagnosis.

## Conclusion

JIA is still a diagnosis of exclusion. We should use modern diagnostic procedures to establish right diagnosis and mustn’t give antirheumatic drugs except NSAID to our patients until the diagnosis is verified.

## Disclosure of interest

None declared.

